# New Criteria for Synchronization of Multilayer Neural Networks via Aperiodically Intermittent Control

**DOI:** 10.1155/2022/8157794

**Published:** 2022-09-27

**Authors:** Taiyan Jing, Daoyuan Zhang, Xiaohua Zhang

**Affiliations:** ^1^School of Mathematics and Information Science, Henan Polytechnic University, Jiaozuo 454000, China; ^2^Department of Artificial Intelligence and Data Science, Guangzhou Xinhua University, Guangzhou 523133, Guangdong Province, China; ^3^Department of Mathematics, Jiangsu Vocational Institute of Commerce, Nanjing 210016, China

## Abstract

In this paper, the globally asymptotic synchronization of multi-layer neural networks is studied via aperiodically intermittent control. Due to the property of intermittent control, it is very hard to deal with the effect of time-varying delays and ascertain the control and rest widths for intermittent control. A new lemma with generalized Halanay-type inequalities are proposed first. Then, by constructing a new Lyapunov–Krasovskii functional and utilizing linear programming methods, several useful criteria are derived to ensure the multilayer neural networks achieve asymptotic synchronization. Moreover, an aperiodically intermittent control is designed, which has no direct relationship with control widths and rest widths and extends existing aperiodically intermittent control techniques, the control gains are designed by solving the linear programming. Finally, a numerical example is provided to confirm the effectiveness of the proposed theoretical results.

## 1. Introduction

In the past few decades, coupled neural networks have drawn much attention because of their inherent characteristics and wide applications, such as secure communication [1], image encryption [2], and information processing [3]. As one of the fundamental research areas, synchronization is used to better understand the self-organization phenomena among coupled systems, which can be existed in many physical, social, and biological systems with various applications [4–6]. From the viewpoint of practical applications, investigating globally asymptotic synchronization of coupled neural networks is meaningful [7, 8].

It is well known that time delays are unavoidable for coupled neural networks due to the limited bit rate of communication channels and the limited bandwidth. Therefore, much attention has been attracted to studying the synchronization problem of coupled neural networks with time delays [9]. Yang et al. [10] investigated the synchronization problem of coupled time-delay neural networks with mode-dependent average dwell time switching. In [11], synchronization of memristive neural networks with mixed delays via quantized intermittent control was considered. However, the aforementioned results of delayed neural networks are based on one or two layers networks. In fact, multilayer neural networks with more than two layers can be seen as some subnetworks distributed in different layers. For example, there exist three-layers networks about information transmission in smart grids [12] and Kuramoto-oscillator networks [13–15]. Therefore, it is necessary to study the globally asymptotic synchronization for multilayer dynamic networks with time delays.

To drive dynamic networks to achieve synchronization, suitable controllers should be designed and added to the nodes of dynamic networks. In practical applications, the transmitted information is inevitably affected by external perturbations, which make the information weak or interrupted intermittently. In this case, the continuous-time control is not suitable. Hence, intermittent control schemes were proposed [16, 17]. Moreover, intermittent control can greatly reduce control cost and the amount of transmitted signals. Considering the fact that the structural limitation of periodically intermittent control is not applicable in reality [18], aperiodically intermittent control was considered in [19, 20], which is characterized by nonfixed control time and rest time in a nonfixed time span. In [21], Liu et al. investigated the exponential synchronization problem for linearly coupled networks with delay by using aperiodically intermittent control. The authors of [22, 23] considered finite-time synchronization of delayed dynamic networks via aperiodically intermittent control. However, the above-given existing aperiodically intermittent control is complex and some strict restrictions are imposed on the control width and noncontrol width, which make it difficult to be implemented in practice. It is demonstrated that new aperiodically intermittent control methods [24] are proposed to improve the above existing control. Thus, adopting a new control strategy to study the asymptotic synchronization of multilayer delayed networks is necessary.

Motivated by the above-given analysis, this paper is devoted to studying the asymptotic synchronization of multilayer delayed networks by aperiodically intermittent control. The main contributions are summarized as follows:

1. An original lemma is an extended form of many general Halanay-type differential inequalities [21, 25–27]. The lemma is proposed for the asymptotic stability with an intermittent divergence of system's state and is applicable to the asymptotic synchronization of delayed networks with intermittent control.
2. Aperiodically intermittent controllers without any information of time delays are designed, which need less restrictive conditions and make the controllers more economic and practical than those controllers in [21–23].
3. Novel Lyapunov–Krasovskii functional is designed, which can reduce the conservativeness of the results. Sufficient conditions derived by linear programming methods are acquired to ensure the asymptotic synchronization of delayed networks with intermittent control, where the effects of time delays are well dealt with.

The rest of this paper is organized as follows: in Section 2, some necessary assumptions and lemmas are given. In Section 3, the asymptotic synchronization of multilayer delayed neural networks is studied via aperiodically intermittent control. In Section 4, a numerical example is given to verify the effectiveness of the proposed theoretical schemes. Conclusions are drawn in Section 5.

Notations: let *ℝ*^*n*^ denote the *n*-dimensional Euclidean space and *ℝ*^*n*×*m*^ denote the set of *n* × *m* real matrix. ⊗ stands for Kronecker product. *a*_*i*_(*t*), *b*_*i*_(*t*)(*i*=1,2,3,4)(*t* ∈ *ℝ*) are continuous and bounded functions. *a*_*i∗*_=inf_*t*∈*ℝ*_*a*_*i*_(*t*), *a*_*i*_^*∗*^=sup_*t*∈*ℝ*_*a*_*i*_(*t*), *b*_*i∗*_=inf_*t*∈*ℝ*_*b*_*i*_(*t*), *b*_*i*_^*∗*^=sup_*t*∈*ℝ*_*b*_*i*_(*t*), *i*=1,2,3,4.

## 2. Preliminaries

In this paper, we consider the following dynamic networks with *p* layers and *N* nonlinearly identical nodes:

(1) $$\begin{array}{c} {\dot{x}}_{i}\left(t\right)=f_{i}\left(x_{i}\left(t\right)\right)+\overset{N}{\underset{j=1}{\sum }}a_{ij}^{0}g_{0j}\left(x_{j}\left(t\right)\right)+\overset{p-1}{\underset{r=1}{\sum }}\overset{N}{\underset{j=1}{\sum }}a_{ij}^{r}g_{rj}\left(x_{j}\left(t-\tau_{r}\left(t\right)\right)\right)\text{,} \end{array}$$

where *x*_*i*_(*t*)=[*x*_*i*1_(*t*), *x*_*i*2_(*t*),…,*x*_*in*_(*t*)]^*T*^ ∈ *ℝ*^*n*^ denotes the state vector of the *i* th node. The function *f*_*i*_(·) : *ℝ*^*n*^⟶*ℝ*^*n*^ and *g*_*rj*_(·) : *ℝ*^*n*^⟶*ℝ*^*n*^ are continuous.

To simply the notations, let *x*(*t*)=[*x*_1_(*t*)^*T*^,…,*x*_*N*_(*t*)^*T*^]^*T*^, *f*(*x*)=[*f*_1_(*x*_1_),…,*f*_*N*_(*x*_*N*_)]^*T*^ and *g*_*r*_(*x*)=[*g*_*r*1_^*T*^,…,*g*_*rN*_^*T*^]^*T*^(*r*=0,1,…, *p* − 1), *g*_*rj*_(·) : *ℝ*^*n*^⟶*ℝ*^*n*^ are continuous. The functions *τ*_*r*_(*t*) > 0(*r*=1,…, *p* − 1) denote the bounded and continuously differentiable coupling delays, which means there exist positive constants $\tilde{\tau }$ and *ϖ* such that $0<\tau_{r}\left(t\right)\leq \tilde{\tau }$ and $0\leq {\dot{\tau }}_{r}\left(t\right)\leq \varpi <1\left(r=1,\ldots ,p-1\right)$. *A*_*r*_=(*a*_*ij*_^*r*^) ∈ *ℝ*^*N*×*N*^(*r*=0,1,…, *p* − 1) are the weight configuration matrices. If there is a link between nodes *i* and *j*(*i* ≠ *j*), then *a*_*ij*_^*r*^=*a*_*ji*_^*r*^ > 0; otherwise, *a*_*ij*_^*r*^=*a*_*ji*_^*r*^=0, and the diagonal elements of matrices *A*_*r*_ are represented by the following equation:

(2) $$\begin{array}{c} a_{ii}^{r}=-\overset{N}{\underset{j=1,j\neq i}{\sum }}a_{ij}^{r},r=0,1,\ldots ,p-1\text{.} \end{array}$$

For simplicity, the drive system (5) can be written in the Kronecker product form:

(3) $$\begin{array}{c} \dot{x}\left(t\right)=f\left(x\left(t\right)\right)+\left(A_{0}⊗I_{n}\right)g_{0}\left(x\left(t\right)\right)+\overset{p-1}{\underset{r=1}{\sum }}\left(A_{r}⊗I_{n}\right)g_{r}\left(x\left(t-\tau_{r}\left(t\right)\right)\right)\text{,} \end{array}$$

The corresponding response systems are written as follows:

(4) $$\begin{array}{c} \dot{y}\left(t\right)=f\left(y\left(t\right)\right)+\left(A_{0}⊗I_{n}\right)g_{0}\left(y\left(t\right)\right)+\overset{p-1}{\underset{r=1}{\sum }}\left(A_{r}⊗I_{n}\right)g_{r}\left(y\left(t-\tau_{r}\left(t\right)\right)\right)+u\left(t\right)\text{,} \end{array}$$

where *y*(*t*)=(*y*_1_^*T*^(*t*),…,*y*_*N*_^*T*^(*t*))^*T*^, *y*_*i*_(*t*)=[*y*_*i*1_(*t*), *y*_*i*2_(*t*),…,*y*_*in*_(*t*)]^*T*^ ∈ *ℝ*^*n*^ denotes the response output vector of the *i* th node. *u*(*t*)=[*u*_1_^*T*^(*t*),…,*u*_*N*_^*T*^(*t*)]^*T*^, *u*_*i*_(*t*) denotes the input control of the *i* th node.

**Remark 1 .**

When the multi-layer parameter *p*=2, the same models (3) degenerate into that considered in [23], even many similar models to (3) were discussed, e.g., [21, 22]. Therefore, the models of systems (3) in this paper are broader model forms. In addition, most of the practical neural networks are interrelated and interact with each other such that they generate more complicated structures and unpredictable behaviors than that with one layer. That is, general models with multilayer structures can simulate the real network world better, please see, e.g., [28, 29]. Thus, the models are worthy to be further discussed.

In this paper, the structure of aperiodically intermittent control is briefly described as follows: each time span [*t*_2*k*_, *t*_2*k*+1_) and [*t*_2*k*+1_, *t*_2*k*+2_)(*t*_0_=*t*_−1_=*t*_−2_=0, *k*=−1,0,1,2,…) denote the control time and the rest time, respectively. The aperiodically intermittent control becomes a periodic one, when *t*_2*k*+2_ − *t*_2*k*_ ≡ *T* , *t*_2*k*+1_ − *t*_2*k*_ ≡ *δ*, where *T*, *δ* are positive constants and 0 < *δ* < *T*.

The main objective is to apply the aperiodically intermittent control to force the states of networks (4) to be asymptotically synchronized with the ones of (3), i.e., lim_*t*⟶*∞*_‖*y*(*t*) − *x*(*t*)‖=0. The multilayer error systems *e*(*t*)=*y*(*t*) − *x*(*t*) are obtained as follows:

(5) $$\begin{array}{c} \dot{e}\left(t\right)=f\left(y\left(t\right)\right)-f\left(x\left(t\right)\right)+\left(A_{0}⊗I_{n}\right)g_{0}\left(y\left(t\right)\right)-\left(A_{0}⊗I_{n}\right)g_{0}\left(x\left(t\right)\right)+\overset{p-1}{\underset{r=1}{\sum }}\left(A_{r}⊗I_{n}\right)g_{r}\left(y\left(t-\tau_{r}\left(t\right)\right)\right)-\overset{p-1}{\underset{r=1}{\sum }}\left(A_{r}⊗I_{n}\right)g_{r}\left(x\left(t-\tau_{r}\left(t\right)\right)\right)+u\left(t\right)\text{.} \end{array}$$

To obtain our main results, the following assumptions and lemmas are given as follows.

**Assumption 1 (see [30]).**

If there exists a positive definite diagonal matrix *P*=diag{*P*_1_, *P*_2_,…, *P*_*N*_} ∈ *ℝ*^*nN*×*nN*^, a diagonal matrix Δ=diag{Δ_1_, Δ_2_,…, Δ_*N*_} ∈ *ℝ*^*nN*×*nN*^, and a positive scalar *ξ* such that

(6) $$\begin{array}{c} {\left(y\left(t\right)-x\left(t\right)\right)}^{T}P\left[f\left(y\left(t\right)\right)-f\left(x\left(t\right)\right)-\Delta \left(y\left(t\right)-x\left(t\right)\right)\right]\leq -\xi {\left(y\left(t\right)-x\left(t\right)\right)}^{T}\left(y\left(t\right)-x\left(t\right)\right)\text{,} \end{array}$$

holds for any *x*(*t*), *y*(*t*) ∈ *ℝ*^*nN*^, where *P*_*i*_, Δ_*i*_ ∈ *ℝ*^*n*×*n*^ are diagonal matrices.

**Lemma 1 (see [31]).**

Given any real matrices *X*, *Y* and *K* of appropriate dimensions and a scalar *ε* > 0 such that *K*=*K*^*T*^ > 0. Then, the following inequality holds:

(7) $$\begin{array}{c} X^{T}Y+Y^{T}X\leq \varepsilon X^{T}KX+\varepsilon^{-1}Y^{T}K^{-1}Y\text{.} \end{array}$$

Obviously, when *K*=*I* (*I* is an identity matrix), the inequality is transformed into *X*^*T*^*Y*+*Y*^*T*^*X* ≤ *εX*^*T*^*X*+*ε*^−1^*Y*^*T*^*Y*.

**Lemma 2 (see [32]).**

Let $\tilde{x}\left(\cdot \right)$ be a nonnegative function satisfying the following equation:

(8) $$\begin{array}{c} \left\{\begin{array}{c} \dot{\tilde{x}}\left(t\right)\leq -a\left(t\right)\tilde{x}\left(t\right)+b\left(t\right)\left(\underset{t-\tau \left(t\right)\leq s\leq t}{sup}\tilde{x}\left(s\right)\text{,}\right)\;t>t_{0}\text{,}\\ \tilde{x}\left(s\right)=\left|\varphi \left(s\right)\right|,\forall s\in \left[t_{0}-\tau^{*},t_{0}\right]\text{,} \end{array}\right. \end{array}$$

where *τ*(*t*) denotes a nonnegative, continuous and bounded function defined for *t* ∈ *ℝ* and $\tau^{*}=\underset{t\in \mathbb{R}}{sup}\tau \left(s\right)$; *φ*(*s*) is continuous and defined for *s* ∈ [*t*_0_ − *τ*^*∗*^, *t*_0_]; *a*(*t*) and *b*(*t*)(*t* ∈ *ℝ*) denote nonnegative, continuous, and bounded functions. Suppose

(9) $$\begin{array}{c} a\left(t\right)-b\left(t\right)\geq \sigma ,t\in R\text{,} \end{array}$$

where $\sigma =\underset{t\in \mathbb{R}}{inf}\left(a\left(t\right)-b\left(t\right)\right)>0$. Let $0<\tilde{\sigma }<\sigma$, there exists a positive number $\tilde{\mu }$ satisfying the following inequality:

(10) $$\begin{array}{c} -a\left(t\right)+\tilde{\mu }+b\left(t\right)\exp\;\left\{\tilde{\mu }\tau^{*}\right\}\leq -\tilde{\sigma }<0,\text{for all}t\in R\text{.} \end{array}$$

Then,

(11) $$\begin{array}{c} \tilde{x}\left(t\right)\leq \left(\underset{s\in \left[t_{0}-\tau^{*},t_{0}\right]}{sup}\tilde{x}\left(s\right)\right)\exp\;\left\{-\tilde{\mu }\left(t-t_{0}\right)\right\},t>t_{0}\text{.} \end{array}$$

**Lemma 3 (see [33]).**

Let $\left.w\left(\cdot \right):\left.\left[t_{0}-\bar{\tau },+\infty \right.\right)⟶\left[0,+\infty \right.\right)$ be a continuous function such that $\dot{w}\left(t\right)\leq a\left(t\right)w\left(t\right)+b\left(t\right)\bar{w}\left(t\right)$ holds for *t* ≥ *t*_0_, where $\bar{w}\left(t\right)={sup}_{-\bar{\tau }\leq k\leq 0}\;\left(w\left(t+k\right)\right)$. If *b*(*t*) > 0 and *a*(*t*)+*b*(*t*) ≥ *m*^*∗*^, we have the following equation:

(12) $$\begin{array}{c} w\left(t\right)\leq \bar{w}\left(t_{0}\right)\exp\;\left\{m^{*}\left(t-t_{0}\right)\right\},t\geq t_{0}\text{.} \end{array}$$

It should be noted that realizing the asymptotic synchronization is generally difficult due to the use of intermittent control and the effect of time-varying delay. the intermittent control shows some difficulties to be handled, including the fact that the values of error state *e*(*t*)=*y*(*t*) − *x*(*t*) increase on all rest intervals. The time-varying delay brings several uncertain factors in tending towards the process of asymptotic synchronization. However, these difficulties will be well dealt with by studying new analytical methods.

Before proceeding with our research, a lemma is given in the following, with which the difficulty induced by intermittent control is surmounted.

**Lemma 4 .**

Assume that a function $\tilde{y}\left(t\right)$ is continuous and nonnegative when $t\in \left(-\tilde{\tau },+\infty \right)$, and satisfies the following condition:

(13) $$\begin{array}{c} \left\{\begin{array}{c} \dot{\tilde{y}}\left(t\right)\leq -a_{1}\left(t\right)\tilde{y}\left(t\right)+a_{2}\left(t\right){sup}_{0<\tau_{r}\left(t\right)\leq \tilde{\tau },1\leq r\leq p-1}\tilde{y}\left(t-\tau_{r}\left(t\right)\right),t_{2k}\leq t<t_{2k+1}\text{,}\\ \dot{\tilde{y}}\left(t\right)\leq a_{3}\left(t\right)\tilde{y}\left(t\right)+a_{4}\left(t\right){sup}_{0<\tau_{r}\left(t\right)\leq \tilde{\tau },1\leq r\leq p-1}\tilde{y}\left(t-\tau_{r}\left(t\right)\right),t_{2k+1}\leq t<t_{2k+2}\text{,} \end{array}\right. \end{array}$$

where *k* ∈ *ℕ* and *p* > 1 is a positive integer. Assume further that the following inequalities hold, i.e.,

(14) $$\begin{array}{c} \underset{t\in R}{sup}\left\{a_{1}\left(t\right)-a_{2}\left(t\right)\right\}=\lambda >0,a_{3}\left(t\right)+a_{4}\left(t\right)\geq \varepsilon >0,\frac{\lambda \left(t_{2k+1}-t_{2k}\right)}{\varepsilon \left(t_{2k+2}-t_{2k+1}\right)}=\chi_{k}>1\text{.} \end{array}$$

Then, $\tilde{y}\left(t\right)⟶0$ as *t*⟶+*∞*.

**Proof**

Denote $\bar{y}={sup}_{s\in \left[t_{0}-\tilde{\tau },t_{0}\right]}\tilde{y}\left(s\right)$, then from Lemma 2, when *t* ∈ [*t*_0_, *t*_1_), it is obtained from that

(15) $$\begin{array}{c} \tilde{y}\left(t\right)\leq \bar{y}\exp\;\left\{-\lambda \left(t-t_{0}\right)\right\}\text{,}\\ \tilde{y}\left(t_{1}\right)\leq \bar{y}\exp\;\left\{-\lambda \left(t_{1}-t_{0}\right)\right\}\text{,} \end{array}$$

where *t*_0_=0.

From Lemma 3, when *t* ∈ [*t*_1_, *t*_2_), the second inequality of (13) leads to the following inequality.

(16) $$\begin{array}{c} \tilde{y}\left(t\right)\leq \tilde{y}\left(t_{1}\right)\exp\;\left\{\varepsilon \left(t-t_{1}\right)\right\}\text{,} \end{array}$$

which means that

(17) $$\begin{array}{c} \tilde{y}\left(t_{2}\right)\leq \tilde{y}\left(t_{1}\right)\exp\;\left\{\varepsilon \left(t_{2}-t_{1}\right)\right\}\text{.} \end{array}$$

Similarly, when *t* ∈ [*t*_2_, *t*_3_), one has

(18) $$\begin{array}{c} \tilde{y}\left(t\right)\leq \tilde{y}\left(t_{2}\right)\exp\;\left\{-\lambda \left(t-t_{2}\right)\right\}\leq \bar{y}\exp\;\left\{-\lambda \left(t_{1}-t_{0}\right)\right\}\exp\;\left\{\varepsilon \left(t_{2}-t_{1}\right)\right\}\exp\;\left\{-\lambda \left(t-t_{2}\right)\right\}\text{,} \end{array}$$

and

(19) $$\begin{array}{c} \tilde{y}\left(t_{3}\right)\leq \bar{y}\exp\;\left\{-\lambda \left(t_{1}-t_{0}\right)\right\}\exp\;\left\{\varepsilon \left(t_{2}-t_{1}\right)\right\}\exp\;\left\{-\lambda \left(t_{3}-t_{2}\right)\right\}=\bar{y}\exp\;\left\{-\lambda \left[\left(t_{1}-t_{0}\right)+\left(t_{3}-t_{2}\right)\right]\right\}\exp\;\left\{\varepsilon \left(t_{2}-t_{1}\right)\right\}\text{.} \end{array}$$

By induction, when *t* ∈ [*t*_2*k*_, *t*_2*k*+1_), *k* ∈ *ℕ*^+^, it follows that

(20) $$\begin{array}{c} \tilde{y}\left(t\right)\leq \bar{y}\exp\;\left\{-\lambda \overset{k-1}{\underset{m=-1}{\sum }}\left(t_{2m+1}-t_{2m}\right)\right\}\exp\;\left\{\varepsilon \overset{k}{\underset{m=0}{\sum }}\left(t_{2m}-t_{2m-1}\right)\right\}\exp\;\left\{-\lambda \left(t-t_{2k}\right)\right\}\text{,} \end{array}$$

where *t*_−1_=*t*_−2_=0.

When *t* ∈ [*t*_2*k*+1_, *t*_2*k*+2_), *k* ∈ *ℕ*^+^, it follows that

(21) $$\begin{array}{c} \tilde{y}\left(t\right)\leq \bar{y}\exp\;\left\{-\lambda \overset{k}{\underset{m=0}{\sum }}\left(t_{2m+1}-t_{2m}\right)\right\}\exp\;\left\{\varepsilon \overset{k}{\underset{m=0}{\sum }}\left(t_{2m}-t_{2m-1}\right)\right\}\exp\;\left\{\varepsilon \left(t-t_{2k+1}\right)\right\}\text{.} \end{array}$$

Then, for any *k* ≥ 1, one has from

(22) $$\begin{array}{c} \tilde{y}\left(t\right)\leq \bar{y}\exp\;\left\{-\lambda \overset{k-1}{\underset{m=-1}{\sum }}\left(t_{2m+1}-t_{2m}\right)\right\}\exp\;\left\{\varepsilon \overset{k}{\underset{m=0}{\sum }}\left(t_{2m}-t_{2m-1}\right)\right\}\exp\;\left\{-\lambda \left(t-t_{2k}\right)\right\}=\bar{y}\exp\;\left\{-\lambda \left(t-t_{2k}\right)\right\}\exp\\ \left\{-\lambda \overset{k-1}{\underset{m=0}{\sum }}\left(t_{2m+1}-t_{2m}\right)+\varepsilon \overset{k-1}{\underset{m=0}{\sum }}\left(t_{2m+2}-t_{2m+1}\right)\right\}=\bar{y}\exp\;\left\{-\lambda \left(t-t_{2k}\right)\right\}\exp\;\left\{\int \overset{k-1}{\underset{m=0}{\sum }}\left(1-\chi_{k}\right)\left(t_{2m+2}-t_{2m+1}\right)\right\},t\in \left.\left[t_{2k},t_{2k+1}\right.\right)\text{,}\\ \tilde{y}\left(t\right)\leq \bar{y}\exp\;\left\{-\lambda \overset{k}{\underset{m=0}{\sum }}\left(t_{2m+1}-t_{2m}\right)\right\}\exp\;\left\{\varepsilon \overset{k}{\underset{m=0}{\sum }}\left(t_{2m}-t_{2m-1}\right)\right\}\exp\;\left\{\varepsilon \left(t-t_{2k+1}\right)\right\}\\ \leq \bar{y}\exp\;\left\{-\lambda \left(t_{2k+1}-t_{2k}\right)\right\}\exp\;\left\{-\lambda \overset{k-1}{\underset{m=0}{\sum }}\left(t_{2m+1}-t_{2m}\right)\right\}\exp\;\left\{\varepsilon \overset{k}{\underset{m=0}{\sum }}\left(t_{2m}-t_{2m-1}\right)+\varepsilon \left(t-t_{2k+1}\right)\right\}\\ \leq \bar{y}\exp\;\left\{-\lambda \left(t_{2k+1}-t_{2k}\right)+\varepsilon \left(t_{2k+2}-t_{2k+1}\right)\right\}\exp\;\left\{-\lambda \overset{k-1}{\underset{m=0}{\sum }}\left(t_{2m+1}-t_{2m}\right)\leq \bar{y}\exp\;\left\{-\lambda \left(t_{2k+1}-t_{2k}\right)+\;\varepsilon \left(t_{2k+2}-t_{2k+1}\right)\right\}\exp\;+\varepsilon \overset{k-1}{\underset{m=0}{\sum }}\left(t_{2m+2}-t_{2m+1}\right)\left\{-\lambda \overset{k-1}{\underset{m=0}{\sum }}\left(t_{2m+1}-t_{2m}\right)\varepsilon \overset{k}{\underset{m=1}{\sum }}\left(t_{2m}-t_{2m-1}\right)\right\}\right\}\\ =\bar{y}\exp\;\left\{\varepsilon \overset{k}{\underset{m=0}{\sum }}\left(1-\chi_{k}\right)\left(t_{2m+2}-t_{2m+1}\right)\right\},t\in \left.\left[t_{2k+1},t_{2k+2}\right.\right)\text{.} \end{array}$$

For any *t* ≥ *t*_2_, there is a $\bar{k}\in {\mathbb{N}}_{+}$ such that $\left.t\in \left[t_{2\bar{k}},t_{2\bar{k}+1}\right.\right)$ or $\left.t\in \left[t_{2\bar{k}+1},t_{2\bar{k}+2}\right.\right)$. When *t*⟶+*∞*, it follows that $\sum_{m=0}^{\bar{k}-1}\left(t_{2m+2}-t_{2m+1}\right)⟶+\infty$. Since *χ*_*k*_ > 1, one has $\exp\;\left\{\varepsilon \sum_{m=0}^{\bar{k}-1}\left(1-\chi_{k}\right)\left(t_{2m+2}-t_{2m+1}\right)\right\}⟶0$ and $\exp\;\left\{\varepsilon \sum_{m=0}^{\bar{k}}\left(1-\chi_{k}\right)\left(t_{2m+2}-t_{2m+1}\right)\right\}⟶0$. To sum up, $\tilde{y}\left(t\right)⟶0$ as *t*⟶+*∞*. The proof is completed.

**Remark 2 .**

When the coefficients of differential inequalities (13) become constants (i.e., *a*_*i*_(*t*)=*a*_*i*_, *i*=1,2,3, *a*_4_(*t*)=*a*_2_) and the layers become one layer (*p*=2), this lemma degenerates into the one studied in [21].  Lemma 4 relaxes the limiting conditions of the coefficients in the inequalities and generalizes the differential form of the inequalities. Moreover, without the existence of intermittent, Lemma 4 degenerates into Lemma 2 in [32] and Lemma 3 in [33]. Thus, Lemma 4 is a more general form and can be applied to the case of both nonintermittent and intermittent.

**Remark 3 .**

Lemma 4 can be applied to asymptotic synchronization or asymptotic stabilization via aperiodically intermittent control. Compared with the conditions of the designed aperiodically intermittent control in [21, 22], Lemma 4 relaxes some harsh conditions. For example, in [23], the lower bound of control interval (i.e., inf_*k*∈*ℕ*_ {*t*_2*k*+1_ − *t*_2*k*_}=*θ*) and the upper bound of rest width (i.e., inf_*k*∈*ℕ*_ {*t*_2*k*+2_ − *t*_2*k*+1_}=*ω*) have to be previously assumed and satisfied *θ* < *ω*, where *θ* and *ω* are two positive constants. It presents from the condition (*λ*(*t*_2*k*+1_ − *t*_2*k*_)/*ε*(*t*_2*k*+2_ − *t*_2*k*+1_))=*χ*_*k*_ that the intervals of control and rest can be flexibly adjusted. That is, both the control interval and the rest interval can be arbitrarily large. Therefore, the aperiodically intermittent controller shows its superiority and operability in practice.

## 3. Synchronization of Multilayer Neural Networks with Time-Varying Delays via Aperiodically Intermittent Control

This section is aimed to investigate the asymptotic synchronization problem of multi-layer neural networks with time-varying delays via aperiodically intermittent control. With the help of designing strictly aperiodically intermittent control and applying Lemma 4, Theorem 1 is obtained to ensure that multilayer neural networks (4) asymptotically synchronize on (3).

Design a mode-dependent controller as follows:

(23) $$\begin{array}{c} u\left(t\right)=\left\{\begin{array}{cc} -De\left(t\right), & t_{2k}\leq t<t_{2k+1}\text{,}\\ 0\text{,} & t_{2k+1}\leq t<t_{2k+2}\text{,} \end{array}\right. \end{array}$$

where *e*(*t*)=[*e*_1_^*T*^(*t*),…,*e*_*N*_^*T*^(*t*)]^*T*^, *e*_*i*_(*t*)=*y*_*i*_(*t*) − *x*_*i*_(*t*)(*i*=1,2,…, *n*), *D*=diag(*d*_1_, *d*_2_,…, *d*_*nN*_), and *d*_*i*_ > 0 are the control gains.

**Remark 4 .**

To eliminate the effects of time delays, some complex terms, such as integrals and time delays, are added to the intermittent controller in [34]. However, in many cases, it is difficult to obtain detailed information of time delays; hence, intermittent controller with time delays is usually difficult to be implemented in practice. Therefore, an intermittent controller without any information of delays is designed, which makes the controller more practical than the state-feedback controller in [35].

For the above-designed controller, we will reveal how to design suitable control gains *d*_*i*_(*i*=1,2,…, *nN*) such that the multilayer error systems (5) can achieve asymptotic synchronization. The main results are elaborated as follows.

**Theorem 1 .**

Assume that the function *f*(*t*) satisfies Assumption 1 and the function *g*(*t*) satisfies the Lipschitz condition, there exist positive constants *ξ*, *ξ*_1_, *ξ*_2_, *l*, *b*_1_^*∗*^, *b*_3*∗*_, *ϖ*, *ς*, *d*_*i*_(*i*=1,2,…, *nN*) such that

- $\left(b_{1}^{*}/2\right)P-\xi I_{nN}+P\Delta +\xi_{1}{\left(A_{0}⊗I_{n}\right)}^{T}P\left(A_{0}⊗I_{n}\right)+\left(l^{2}/\xi_{1}\right)P+\sum_{r=1}^{p-1}\xi_{2}{\left(A_{r}⊗I_{n}\right)}^{T}P\left(A_{r}⊗I_{n}\right)-D+\exp\;\left\{b_{1}^{*}\tilde{\tau }\right\}/2\left(p-1\right)P<0$,
$-\left(1/2\right)b_{3*}P-\xi I_{nN}+P\Delta +\xi_{1}{\left(A_{0}⊗I_{n}\right)}^{T}P\left(A_{0}⊗I_{n}\right)+\left(l^{2}/\xi_{1}\right)I_{nN}+\sum_{r=1}^{p-1}\xi_{2}{\left(A_{r}⊗I_{n}\right)}^{T}P\left(A_{r}⊗I_{n}\right)+\left(\exp\;\left\{b_{1}^{*}\tilde{\tau }\right\}/2\right)\left(p-1\right)P<0$,
- $b_{2}\left(t\right)=2\left(p-1\right)\left(\left(l^{2}/\xi_{2}\right)-\left(\left(1-\varpi \right)\exp\;\left\{b_{1}^{*}\left(\tilde{\tau }-\tau \left(t\right)\right)\right\}/2\right)\right)\geq 0$,

where *p* > 1 is a positive integer, the matrixes *P*, Δ ∈ *ℝ*^*nN*^ are defined in Assumption 1. Assume further that the inequalities hold, i.e.,

(24) $$\begin{array}{c} \underset{t\in R}{sup}\left\{b_{1}\left(t\right)-b_{2}\left(t\right)\right\}=\mu >0,b_{3}\left(t\right)+b_{4}\left(t\right)\geq \nu >0,\frac{\mu \left(t_{2k+1}-t_{2k}\right)}{\nu \left(t_{2k+2}-t_{2k+1}\right)}=\chi_{k}>1\text{.} \end{array}$$

Then, the multilayer error systems (5) are globally asymptotically synchronized.

**Proof**

Consider the following Lyapunov–Krasovskii functional:

(25) $$\begin{array}{c} V\left(t\right)=V_{1}\left(t\right)+V_{2}\left(t\right)\text{,} \end{array}$$

where *V*_1_(*t*)=1/2*e*^*T*^(*t*)*Pe*(*t*), $V_{2}\left(t\right)=\exp\;\left\{b_{1}^{*}\tilde{\tau }\right\}/2\sum_{r=1}^{p-1}\int_{t-\tau_{r}\left(t\right)}^{t}\exp\;\left\{b_{1}^{*}\left(s-t\right)\right\}e^{T}\left(s\right)Pe\left(s\right)ds$.

Computing the derivative of *V*(*t*), we have the following equation:

(26) $$\begin{array}{c} {\dot{V}}_{1}\left(t\right)=-b_{1}\left(t\right)V_{1}\left(t\right)+\frac{b_{1}\left(t\right)}{2}e^{T}\left(t\right)Pe\left(t\right)+e^{T}\left(t\right)P\dot{e}\left(t\right)\text{,} \end{array}$$

(27) $$\begin{array}{c} {\dot{V}}_{2}\left(t\right)=-b_{1}^{*}V_{2}\left(t\right)+\frac{\exp\;\left\{b_{1}^{*}\tilde{\tau }\right\}}{2}\left[\left(p-1\right)e^{T}\left(t\right)Pe\left(t\right)-\overset{p-1}{\underset{r=1}{\sum }}\exp\;\left\{-b_{1}^{*}\tau_{r}\left(t\right)\right\}\times \left(1-\tau_{r}\left(t\right)\right)e^{T}\left(t-\tau_{r}\left(t\right)\right)Pe\left(t-\tau_{r}\left(t\right)\right)\right] \end{array}$$

From (26) and (27), along the trajectories of (5) with controllers (23), we deduce two cases as follows.

**Case 1 .**

When *t*_2*k*_ ≤ *t* < *t*_2*k*+1_, for *k* ∈ *ℕ*

(28) $$\begin{array}{c} \dot{V}\left(t\right)\leq -b_{1}\left(t\right)V\left(t\right)+\frac{b_{1}\left(t\right)}{2}e^{T}\left(t\right)Pe\left(t\right)+e^{T}\left(t\right)P\left\{\left[f\left(y\left(t\right)\right)-f\left(x\left(t\right)\right)\right]+\left(A_{0}⊗I_{n}\right)\left[g_{0}\left(y\left(t\right)\right)-g_{0}\left(x\left(t\right)\right)\right]+\overset{p-1}{\underset{r=1}{\sum }}\left(A_{r}⊗I_{n}\right)\left[g_{r}\left(y\left(t-\tau_{r}\left(t\right)\right)\right)-g_{r}\left(x\left(t-\tau_{r}\left(t\right)\right)\right)\right]-De\left(t\right)\right\}\\ +\frac{\exp\;\left\{b_{1}^{*}\tilde{\tau }\right\}}{2}\left[\left(p-1\right)e^{T}\left(t\right)Pe\left(t\right)-\overset{p-1}{\underset{r=1}{\sum }}\exp\;\left\{-b_{1}^{*}\tau_{r}\left(t\right)\right\}\left(1-\varpi \right)e^{T}\left(t-\tau_{r}\left(t\right)\right)Pe\left(t-\tau_{r}\left(t\right)\right)\right]\text{.} \end{array}$$

Since the function *f*(*t*) satisfies Assumption 1 and the function *g*(*t*) satisfies the Lipschitz condition, and by Lemma 2, we get the following equation:

(29) $$\begin{array}{c} e^{T}\left(t\right)P\left[f\left(y\left(t\right)\right)-f\left(x\left(t\right)\right)-\Delta e\left(t\right)\right]\leq -\xi e^{T}\left(t\right)e\left(t\right),e^{T}\left(t\right)P\left(A_{0}⊗I_{n}\right)\left[g_{0}\left(y\left(t\right)\right)-g_{0}\left(x\left(t\right)\right)\right]\leq \xi_{1}e^{T}\left(t\right){\left(A_{0}⊗I_{n}\right)}^{T}P\left(A_{0}⊗I_{n}\right)e\left(t\right)+\frac{l^{2}}{\xi_{1}}e^{T}\left(t\right)Pe\left(t\right)\text{,}\\ e^{T}\left(t\right)P\left(A_{r}⊗I_{n}\right)\left[g_{r}\left(y\left(t-\tau_{r}\left(t\right)\right)\right)-g_{r}\left(x\left(t-\tau_{r}\left(t\right)\right)\right)\right]\leq \xi_{2}e^{T}\left(t\right){\left(A_{r}⊗I_{n}\right)}^{T}P\left(A_{r}⊗I_{n}\right)e\left(t\right)+\frac{l^{2}}{\xi_{2}}e^{T}\left(t-\tau_{r}\left(t\right)\right)Pe\left(t-\tau_{r}\left(t\right)\right)\text{,} \end{array}$$

where *l*, *ξ*, *ξ*_1_, *ξ*_2_ > 0 are positive constants.

Then, according to the conditions (i) and (iii), it follows from (28) that

(30) $$\begin{array}{c} \dot{V}\left(t\right)\leq -b_{1}\left(t\right)V\left(t\right)+e^{T}\left(t\right)\left[\frac{b_{1}^{*}}{2}P-\xi I_{nN}+P\Delta +\xi_{1}{\left(A_{0}⊗I_{n}\right)}^{T}P\left(A_{0}⊗I_{n}\right)+\frac{l^{2}}{\xi_{1}}P+\overset{p-1}{\underset{r=1}{\sum }}\xi_{2}{\left(A_{r}⊗I_{n}\right)}^{T}P\left(A_{r}⊗I_{n}\right)-D+\frac{\exp\;\left\{b_{1}^{*}\tilde{\tau }\right\}}{2}\left(p-1\right)P\right]\\ e\left(t\right)+\overset{p-1}{\underset{r=1}{\sum }}e^{T}\left(t-\tau_{r}\left(t\right)\right)\left[\frac{l^{2}}{\xi_{2}}-\frac{\exp\;\left\{b_{1}^{*}\tilde{\tau }\right\}}{2}\exp\;\left\{-b_{1}^{*}\tau_{r}\left(t\right)\right\}\left(1-\varpi \right)\right]\times Pe\left(t-\tau_{r}\left(t\right)\right)\leq -b_{1}\left(t\right)V\left(t\right)+b_{2}\left(t\right)\underset{0<\tau_{r}\left(t\right)\leq \tilde{\tau },1\leq r\leq p-1}{sup}V\left(t-\tau_{r}\left(t\right)\right)\text{,} \end{array}$$

where $b_{2}\left(t\right)=2\left(p-1\right)\left(\left(l^{2}/\xi_{2}\right)-\left(\left(1-\varpi \right)\exp\;\left\{b_{1}^{*}\left(\tilde{\tau }-\tau \left(t\right)\right)\right\}/2\right)\right)$.

**Case 2 .**

when *t*_2*k*+1_ ≤ *t* < *t*_2*k*+2_, *k* ∈ *ℕ*, using the conditions (ii) and (iii), we have the following equation:

(31) $$\begin{array}{c} \dot{V}\left(t\right)\leq -b_{1}\left(t\right)V\left(t\right)+\left(b_{1}\left(t\right)+b_{3}\left(t\right)\right)V\left(t\right)+e^{T}\left(t\right)\left[-\frac{1}{2}b_{3*}P-\xi I_{nN}+P\Delta +\xi_{1}{\left(A_{0}⊗I_{n}\right)}^{T}P\left(A_{0}⊗I_{n}\right)+\frac{l^{2}}{\xi_{1}}P+\overset{p-1}{\underset{r=1}{\sum }}\xi_{2}{\left(A_{r}⊗I_{n}\right)}^{T}P\left(A_{r}⊗I_{n}\right)+\frac{\exp\;\left(b_{1}^{*}\tilde{\tau }\right)}{2}\left(p-1\right)P\right]\\ e\left(t\right)+\overset{p-1}{\underset{r=1}{\sum }}e^{T}\left(t-\tau_{r}\left(t\right)\right)\left[\frac{l^{2}}{\xi_{2}}-\frac{\exp\;\left\{b_{1}^{*}\tilde{\tau }\right\}}{2}\times \exp\;\left\{-b_{1}^{*}\tau_{r}\left(t\right)\right\}\left(1-\varpi \right)+\varsigma \right]Pe\left(t-\tau_{r}\left(t\right)\right)\leq b_{3}\left(t\right)V\left(t\right)+b_{4}\left(t\right){sup}_{0<\tau_{r}\left(t\right)\leq \tilde{\tau },1\leq r\leq p-1}\;V\left(t-\tau_{r}\left(t\right)\right)\text{,} \end{array}$$

where *b*_4_(*t*)=(*p* − 1)(*b*_2_(*t*)+*ς*).

Thus, we get the following equation:

(32) $$\begin{array}{c} \left\{\begin{array}{c} \dot{V}\left(t\right)\leq -b_{1}\left(t\right)V\left(t\right)+b_{2}\left(t\right){sup}_{0<\tau_{r}\left(t\right)\leq \tilde{\tau },1\leq r\leq p-1}\;V\left(t-\tau_{r}\left(t\right)\right),t_{2k}\leq t<t_{2k+1}\text{,}\\ \dot{V}\left(t\right)\leq b_{3}\left(t\right)V\left(t\right)+b_{4}\left(t\right){sup}_{0<\tau_{r}\left(t\right)\leq \tilde{\tau },1\leq r\leq p-1}\;V\left(t-\tau_{r}\left(t\right)\right),t_{2k+1}\leq t<t_{2k+2}\text{.} \end{array}\right. \end{array}$$

By Lemma 4 and condition (24), we obtain *V*(*t*)⟶0 as *t*⟶+*∞*. Therefore, $\lim_{t⟶+\infty }\;\left\|e\left(t\right)\right\|=0$. That is, (4) is globally asymptotically synchronized with (3). The proof is completed.

**Remark 5 .**

From condition (24) one can see that, for fixed value *ν*(*t*_2*k*+2_ − *t*_2*k*+1_), the value of the control interval *t*_2*k*+1_ − *t*_2*k*_ can be decreased when the value *μ* is large as long as *χ*_*k*_ > 1 is satisfied. That is, by tuning the value of the control gain *D* such that the value of *μ* increase or decrease. It can be seen that the conditions of Theorem 1 fully reveal the constraint relationship of parameters *D*, *μ* and *ν*.

**Remark 6 .**

Due to the existence of time delay, it will affect the convergence of the error system. By utilizing the inequality (13) in Lemma 4, a new Lyapunov–Krasovskii functional is designed in the proof of Theorem 1, which makes the effect of time delays to be well handled. With the help of Lemma 4, sufficient criteria by linear programming methods for asymptotic synchronization of the drive-response networks (3) and (4) are derived and the conservativeness of the obtained results can be reduced greatly.

## 4. Numerical Simulations

In this section, an example is given to verify the effectiveness of the proposed results in this paper.

**Example 1 .**

We consider the following multilayer dynamic networks with 10 identical nodes:

(33) $$\begin{array}{c} \dot{x}\left(t\right)=f\left(x\left(t\right)\right)+\left(A_{0}⊗I_{3}\right)g_{0}\left(x\left(t\right)\right)+\overset{2}{\underset{r=1}{\sum }}\left(A_{r}⊗I_{3}\right)g_{r}\left(x\left(t-\tau_{r}\left(t\right)\right)\right)\text{,} \end{array}$$

where

(34) $$\begin{array}{c} f_{i}\left(x_{i}\left(t\right)\right)=\left(\begin{array}{ccc} -a & a & 0\\ b & -1 & 0\\ 0 & 0 & -c \end{array}\right)\left(\begin{array}{c} x_{i1}\left(t\right)\\ x_{i2}\left(t\right)\\ x_{i3}\left(t\right) \end{array}\right)+\left(\begin{array}{c} 0\\ -x_{i1}\left(t\right)x_{i3}\left(t\right)\\ x_{i1}\left(t\right)x_{i2}\left(t\right) \end{array}\right)\text{,}\\ g_{0}\left(x\left(t\right)\right)=\sin\;\left(x\left(t\right)\right)\text{,}\\ g_{1}\left(x\left(t-\tau_{1}\left(t\right)\right)\right)=\sin\;\left(x\left(t-\tau_{1}\left(t\right)\right)\right)\text{,}\\ g_{2}\left(x\left(t-\tau_{2}\left(t\right)\right)\right)=0.3x\left(t\right)\cos\;\left(x\left(t-\tau_{2}\left(t\right)\right)\right)\text{,}\\ A_{0}=\left(\begin{array}{cccccccccc} -4 & 0 & 0 & 1 & 1 & 0 & 0 & 0 & 1 & 1\\ 0 & -4 & 1 & 1 & 0 & 1 & 0 & 1 & 0 & 0\\ 0 & 1 & -5 & 1 & 0 & 1 & 0 & 1 & 1 & 0\\ 1 & 1 & 1 & -6 & 0 & 1 & 1 & 1 & 0 & 0\\ 1 & 0 & 0 & 0 & -3 & 1 & 0 & 0 & 1 & 0\\ 0 & 1 & 1 & 1 & 1 & -7 & 1 & 1 & 1 & 0\\ 0 & 0 & 0 & 1 & 0 & 1 & -3 & 0 & 0 & 1\\ 0 & 1 & 1 & 1 & 0 & 1 & 0 & -6 & 1 & 1\\ 1 & 0 & 1 & 0 & 1 & 1 & 0 & 1 & -5 & 0\\ 1 & 0 & 0 & 0 & 0 & 0 & 1 & 1 & 0 & -3 \end{array}\right)\text{,}\\ A_{1}=\left(\begin{array}{cccccccccc} -7 & 1 & 1 & 0 & 1 & 0 & 1 & 1 & 1 & 1\\ 1 & -5 & 1 & 0 & 1 & 0 & 0 & 1 & 1 & 0\\ 1 & 1 & -5 & 1 & 1 & 1 & 0 & 0 & 0 & 0\\ 0 & 0 & 1 & -3 & 1 & 1 & 0 & 0 & 0 & 0\\ 1 & 1 & 1 & 1 & -6 & 0 & 1 & 0 & 0 & 1\\ 0 & 0 & 1 & 1 & 0 & -5 & 1 & 1 & 0 & 1\\ 1 & 0 & 0 & 0 & 1 & 1 & -4 & 0 & 0 & 1\\ 1 & 1 & 0 & 0 & 0 & 1 & 0 & -4 & 1 & 0\\ 1 & 1 & 0 & 0 & 0 & 0 & 0 & 1 & -3 & 0\\ 1 & 0 & 0 & 0 & 1 & 1 & 1 & 0 & 0 & -4 \end{array}\right)\text{,}\\ A_{2}=\left(\begin{array}{cccccccccc} -6 & 1 & 1 & 0 & 0 & 1 & 0 & 1 & 1 & 1\\ 1 & -4 & 1 & 0 & 0 & 1 & 1 & 0 & 0 & 0\\ 1 & 1 & -6 & 0 & 1 & 0 & 1 & 1 & 1 & 0\\ 0 & 0 & 0 & -3 & 1 & 1 & 1 & 0 & 0 & 0\\ 0 & 0 & 1 & 1 & -4 & 1 & 0 & 1 & 0 & 0\\ 1 & 1 & 0 & 1 & 1 & -7 & 1 & 1 & 0 & 1\\ 0 & 1 & 1 & 1 & 0 & 1 & -5 & 0 & 0 & 1\\ 1 & 0 & 1 & 0 & 1 & 1 & 0 & -5 & 1 & 0\\ 1 & 0 & 1 & 0 & 0 & 0 & 0 & 1 & -4 & 1\\ 1 & 0 & 0 & 0 & 0 & 1 & 1 & 0 & 1 & -4 \end{array}\right)\text{,} \end{array}$$

where the parameters are set as *a*=10, *b*=30, *c*=8/3, the time-varying delays are selected as *τ*_1_(*t*)=0.08 exp(*t*)/(1+exp(*t*)) and *τ*_2_(*t*)=0.04 exp(*t*)/(1+exp(*t*)). Note that, *A*_*i*_(*i*=0,1,2) are randomly generated. The control gains in the controllers (23) are selected as *d*_*i*_=100. We choose *P*_*i*_=*di* *ag*{0.5,0.4,0.2} and Δ_*i*_=diag{50,50,50} as [34], it is easy to verify that satisfies Assumption 1 holds with the parameter *ξ*=43.48. Moreover, the initial values of the multilayer systems are given as follows: *x*(0)=(3+*i*, 5+2*i*, 7+2*i*)^*T*^, *y*(0)=(−2+7*i*, −5+6*i*, −7+8*i*)^*T*^(*i*=1,…, 10).

**Figure 1:**
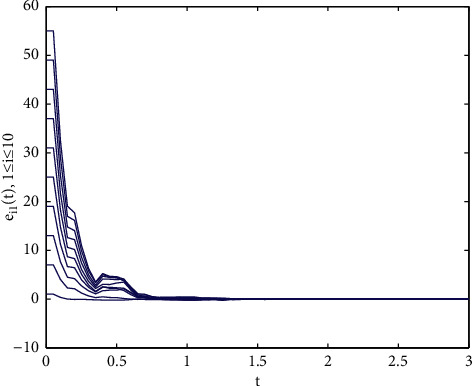
Trajectories of the synchronization errors *e_i_*_1_ roman for number with control gains *d_i_* = 100.

**Figure 2:**
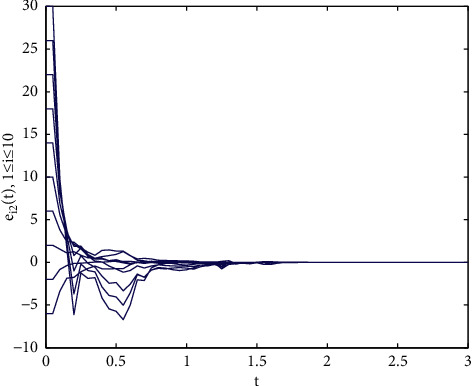
Trajectories of the synchronization errors *e_i_*_2_ with control gains *d_i_* = 100.

**Figure 3:**
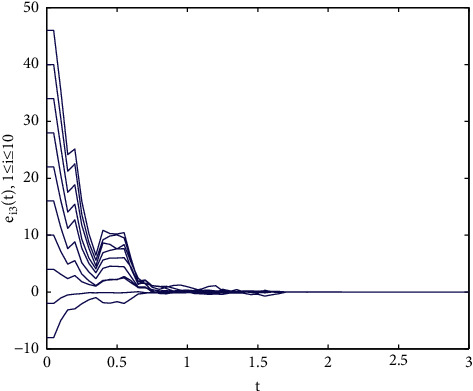
Trajectories of the synchronization errors *e_i_*_3_ with control gains *d_i_* = 100.

**Figure 4:**
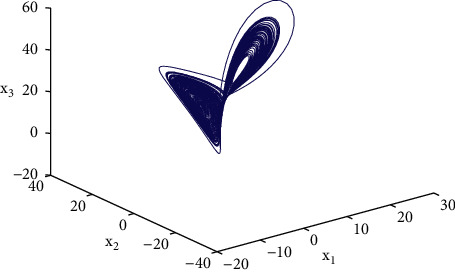
Chaotic attractor of the Lorenz system.

As shown in Figure 1, the Lorenz system ${\dot{x}}_{i}\left(t\right)=f_{i}\left(x_{i}\left(t\right)\right)$ has a chaotic attractor with the initial value *x*(0)=(1,2, −2)^*T*^. Each time span *t*_2*k*+2_ − *t*_2*k*_(*k* ≥ 0) is randomly generated in the interval [0.3*s*, 0.5*s*], and the ratio of the control time *t*_2*k*+1_ − *t*_2*k*_ is randomly generated in the interval [0.5*s*, 0.8*s*], the trajectories of the multilayer system errors with aperiodically intermittent controllers (23) are demonstrated in Figures 2–4.

**Remark 7 .**

To achieve the synchronization effect, we need to set appropriate control parameters *d*_*i*_(*i*=1,2,…, 10) in the controllers (23), which are required to satisfy the conditions in Theorem 1. From these conditions, we can easily see that the upper and lower bounds of each continuous function *b*_*i*_(*t*)(*i*=1, 2, 3, 4) exist, but the concrete values cannot be given. However, we give the sufficiently large parameters *d*_*i*_=100(*i*=1, 2,…, 10) to satisfy condition 1 in Theorem 1.

## 5. Conclusions

In this article, asymptotic synchronization of multilayer neural networks with delays has been studied. By designing strict aperiodically intermittent controllers and establishing a set of novel Halanay-type inequalities, several criteria formulated by linear progressing methods are to ensure asymptotic synchronization. Our results can not only realize asymptotic synchronization of multilayer neural networks with delays by designing strict aperiodically intermittent controllers but also are less conservative in determining the control interval and designing the control gains. Numerical simulation verified the validity of the results obtained here.

At present, by using the aperiodically intermittent control with improved conditions(*λ*(*t*_2*k*+1_ − *t*_2*k*_)/*ε*(*t*_2*k*+2_ − *t*_2*k*+1_)=*χ*_*k*_〉1), many excellent research results on finite-time tracking of uncertain nonlinear systems [36] and finite-time synchronization of delayed neural networks have emerged [11, 19, 20, 24]. Note that, time delay or distributed delay, as one of the vital factors affecting the dynamic behaviors of neural networks, cannot be neglected [8]. In addition, the singularity [37, 38] and fractional-order [39] that constantly appear in practical engineering are the focus of current research. For example, singularly perturbed complex networks with cyberattacks were been considered in [40] and fractional-order nonlinear systems were been discussed in [41]. Therefore, how to realize finite-time synchronization of singularly or fractional-order complex networks with delays or distributed delays is our further research interest, which is also challenging.

## Data Availability

The datasets used and analyzed during the current study are available from the corresponding author upon reasonable request.
